# Advances in Fecal Tests for Colorectal Cancer Screening

**DOI:** 10.1007/s11938-016-0076-0

**Published:** 2016-01-29

**Authors:** Eline H. Schreuders, Esmée J. Grobbee, Manon C. W. Spaander, Ernst J. Kuipers

**Affiliations:** Department of Gastroenterology and Hepatology, Erasmus MC University Medical Center Rotterdam, ‘s-Gravendijkwal 230, 3015 CE Rotterdam, The Netherlands

**Keywords:** Colorectal cancer screening, Fecal immunochemical test, Guaiac fecal occult blood test, DNA testing, Biomarker, FIT, gFOBT

## Abstract

Colorectal cancer (CRC) forms an important public health problem, especially in developed countries. CRC screening tests can be used to identify asymptomatic individuals with CRC precursors and (early) cancer. Removal of these lesions reduces CRC incidence and prevents CRC-related mortality. There are a range of screening tests available, each with advantages and disadvantages. Stool screening tests can broadly be divided into fecal occult blood tests (FOBTs) and molecular biomarker test, such as DNA/RNA marker tests, protein markers, and fecal microbiome marker tests. Guaiac fecal occult blood tests (gFOBT) have been demonstrated in large randomized screening trials to reduce CRC mortality. Fecal immunochemical tests (FIT) have superior adherence, usability, and accuracy as compared to gFOBT. Advantage of the use of quantitative FITs in CRC screening programs is the cut-off level that can be adjusted. Molecular biomarker DNA tests have shown to detect significantly more cancers than FIT. By combining biomarker DNA tests with FIT, sensitivity for advanced adenomas can be increased significantly. However, it has lower specificity thus demands more colonoscopy resources, is more cumbersome, and costly. The adherence has not been assessed in population screening trials. For these reasons, FIT is therefore at present regarded as the preferred method of non-invasive CRC screening. This chapter will review the current status of fecal test-based CRC screening.

## Introduction

Colorectal cancer (CRC) forms an important public health problem, especially in developed countries [1]. It ranks third among the most commonly diagnosed cancers worldwide, affecting approximately 1.23 million patients each year [2]. In developed countries, it is the second cause of cancer-related death in men and the third cause in women [3, 4]. The high incidence and associated mortality, and the natural history of CRC with slow progression from a premalignant polyp to cancer, makes CRC very suitable for population screening [5, 6]. The National Polyp Study within the USA showed that adenoma removal reduced the incidence of CRC by 76–90 %. After a median follow-up of nearly 16 years, colonoscopy with removal of adenomas resulted in a 53 % reduction in CRC mortality (mortality ratio 0.47, 95 % CI 0.26–0.80) compared to the expected CRC mortality rate in the general population [7, 8]. Further studies showed that screening and prevention of CRC is cost-effective and dependent on strategy also cost-saving [9].

Various CRC screening tests are available, which can basically be divided into non-invasive stool or blood tests and more invasive imaging or endoscopy procedures. There is no single worldwide-agreed optimal CRC screening method. This results in different approaches in various countries [10•]. The choice which screening method should be used is mainly dependent on financial and endoscopy resources, and secondly on the willingness of the population to undergo the primary screening test. As a result of limited resources and population preferences for non-invasive screening, many organized screening programs use a two-step approach. This includes primary screening with a non-invasive fecal test, followed by bowel inspection by means of colonoscopy for individuals that tested positive. For a screening test, several test characteristics are necessary. Since screening involves asymptomatic and mostly healthy people, a test should be safe, meaning that the test and screening program should cause no harm. In this light, a high test-specificity is preferred, reducing the risks of harm from both unnecessary (follow-up) testing and overdiagnosis. This is contrary to a diagnostic test in a clinical setting, where the high pretest-probability of the disease has often already been established and the disease needs to be confirmed or ruled out. Furthermore, a screening test should be acceptable to the screenee. Adherence rates of those invited for screening are a direct reflection of the acceptance of the test. A screening program with low adherence will by definition not impact on CRC incidence and mortality, irrespective of the characteristics of the test that is offered. Also, practical use and costs of the test need to be taken into account. Fecal tests may differ in positivity rate, and thus the number of people referred for colonoscopy. They consequently have different demands for colonoscopy resources. Recently, the variability of fecal tests as CRC screening tool is rapidly increasing, and more countries have been implementing CRC screening programs. The large variability and expanding range of fecal tests may impair knowledge of the available screening options. Therefore, this review aims to give an overview of the recent advances in fecal tests and its use in colorectal cancer screening programs.

## Fecal occult blood tests

Fecal occult blood tests (FOBTs) detect hemoglobin (Hb) in feces. A range of FOBTs is available; they can be divided into two types: guaiac FOBT (gFOBT) and fecal immunochemical tests (FIT).

### Guaiac fecal occult blood tests

Guaiac tests were already available a century ago. They were then used to detect gastric blood loss from peptic ulcer and gastric cancer, conditions that affected large numbers of patients. Guaiac FOBTs were in the 1970s the first widely used FOBT for population-based CRC screening. The gFOBT detects blood by the use of guaiac-impregnated paper to which hydro-peroxidase is added. In contact with heme, the hydro-peroxidase oxygenizes guaiac leading to a blue discoloration. The test result, i.e., the blue discoloration, is qualitative (positive/negative). The standard gFOBT consists of three paper cards each with two panels, requiring sampling from three separate stools. Guaiac FOBTs can be analyzed with and without hydration. The former has the advantage of a higher sensitivity; however, it also leads to more false-positives [11]. The impact of gFOBT screening on CRC incidence and mortality has been prospectively assessed in several, large randomized trials. These trials demonstrated that repeated annual or biennial gFOBT screening reduces CRC-related mortality by approximately 32–33 and 6–18 %, respectively [12–15]. The Minnesota trial, which used rehydrated gFOBT, also demonstrated a reduction in CRC incidence [14]. A subsequent meta-analysis reported a pooled 15 % reduction in CRC-related death among the three biennial screening trials with gFOBT compared to controls [16]. The Minnesota trial recently after 30 years follow-up reported an overall 27 % reduction in CRC mortality [17].

A main disadvantage of gFOBT is that it does not specifically target human heme. Hydro-peroxidase also reacts with non-human heme present in red meat. This may cause a false-positive test result. Several fresh fruits and vegetables contain peroxidase activity, which may also lead to false-positive test results. Vitamin C may on the contrary block the peroxidase reaction, resulting in false-negative test results [18]. As a result of the dietary restrictions and the need for three different samples on consecutive days, adherence rates of gFOBT screening are generally poor [18, 19]. Furthermore, although gFOBT has a high specificity, its sensitivity is limited since it does not detect hemoglobin concentrations below approximately 600 μg/g feces [11]. Consequently, adenomatous polyps, precursors of most CRCs, are less likely to be detected as they generally bleed less. The focus on early cancers provides a short window of opportunity, which explains the need for short screening intervals. For these reasons, high sensitivity gFOBTs have been designed, with an enhancer to allow detection of lower Hb concentrations [20]. However, these gFOBTs come with a lower specificity making these test less suitable for population-based screening. Due to the low sensitivity and adherence rates, gFOBT screening is associated with a significant proportion of interval cancers [19]. In the Scottish population program, the proportion of interval cancers increased from 31.2 to 58.9 % after the first, respectively, third screening round [21•]. This increase can partly be explained by a decrease in screen-detected cancers over the screening rounds.

### Fecal immunochemical tests for hemoglobin

**Fig. 1:**
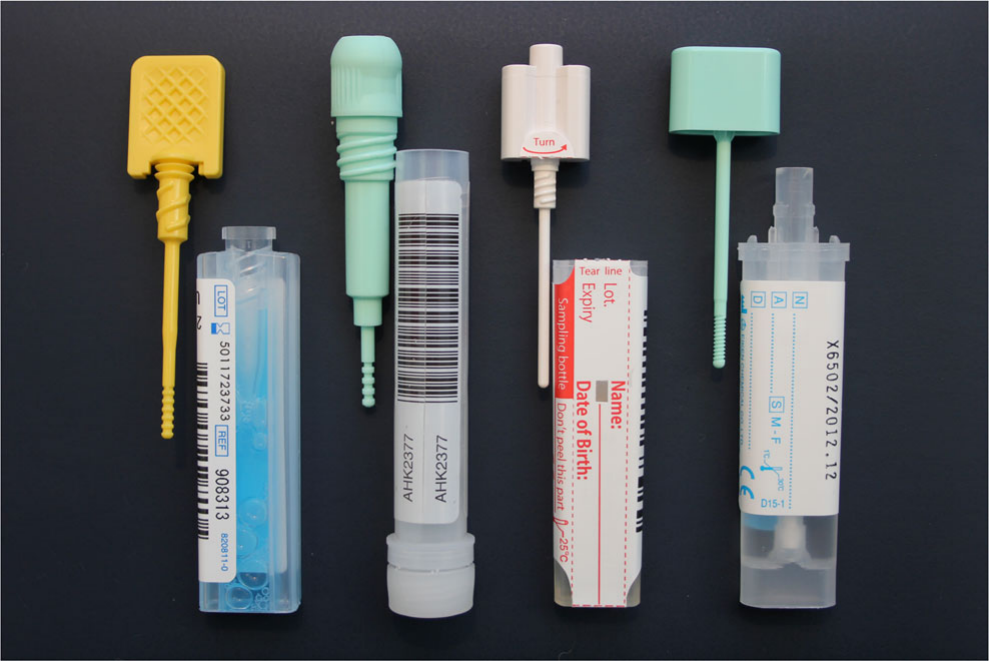
FIT brands with different sampling probes, collection tubes, and volume of preservative buffer.

Fecal immunochemical tests detect human globin by means of an antibody-based assay. FITs either provide a qualitative result or quantitative result in terms of fecal Hb concentration per gram feces. The latter has the advantage that the selection of cut-off level in population-based screening can be tailored to financial and endoscopy resources. There are many different FIT brands available on the market. Figure 1 shows some of the different FITs. These tests sample different amounts of fecal material, use different amounts of buffers, analytical procedures, and reporting units. They generally present results as Hb concentration in nanograms per milliliter test buffer. As a result of these differences, the quantitative results of different tests cannot be compared one-to-one. It has therefore been proposed to standardize the reporting units of fecal Hb to microgram Hb per gram feces [22]. However, even when using these standardized Hb concentrations, different brand of FITs perform differently in mass screening [23]. These differences apply for both qualitative and quantitative results [18]. Currently, there is no evidence for one FIT to be superior over another [19].

At present, there are no results from large prospective randomized trials concerning the impact of repeated FIT screening on CRC incidence and mortality. Even so, the current European guidelines recommend FIT screening as the preferred method of fecal occult blood testing [24, 25]. Screening by means of FIT has advantages over gFOBT screening (Table 1). Firstly, FIT testing requires only one stool sample instead of sampling from three bowel movements. Furthermore, the sampling probe connected to the inside of the lid of the test facilitates test handling (Fig. 1). Together, this results in significantly higher adherence rates with higher detection rates of CRC and advanced adenomas [31]. Also, FIT is more sensitive in detecting hemoglobin than gFOBT with reported sensitivities for advanced neoplasia detection of two to three times higher compared to gFOBT [32]. This higher sensitivity for advanced neoplasia would allow prevention of the development of CRC, and thereby potentially decreasing CRC incidence in addition to detecting CRC in an early stage. Lastly, when comparing FIT to gFOBT regarding cost-effectiveness, FIT screening is more cost-effective at any given cut-off. At the same colonoscopy demand, FIT screening led to lower costs and more life years gained than gFOBT [33].

**Table 1:** Differences between gFOBT and FIT screening in average-risk individuals

gFOBT	FIT
Repeat sampling from multiple bowel movements	Single sampling from one bowel movement
Dietary restrictions	No dietary restrictions
Qualitative result	Quantitative or qualitative result
Semi-automated analysis	Automated analysis
Sensitivity CRC 31 – 63 %^a^	Sensitivity CRC 69 – 100 %^b^
Specificity CRC 92 – 96 %^a^	Specificity CRC 92 – 96 %^b^

^a^[26, 27]

^b^[28–30]

FIT performance shows variability among different subgroups. Some studies reported a higher sensitivity for left-sided adenomas than right-sided lesions [28, 34]. Also, FIT sensitivity has shown to be higher for aspirin users compared with nonusers [35]. At the same cutoff, men have higher FIT positivity rates than women [36]. This is a reflection of the higher prevalence of advanced neoplasia in men, as well as their more frequent distal location [37].

Currently, most quantitative FITs have been mainly used with a fixed cut-off, thereby limiting the FIT to a qualitative result (i.e., either positive or negative). Rationale for choosing a specific cut-off greatly depends on the aim of screening and available colonoscopy resources [33]. Using a higher cut-off is of particular interest in situations with limited colonoscopy capacity where screening programs aim for maximal diagnostic yield with restricted resources [38]. A high cut-off also comes with a high positive predictive value. A lower cut-off increases sensitivity for detection of subjects with advanced neoplasia but requires larger colonoscopy resources due to a lower positive predictive value. There is still much to gain concerning the quantitative nature of FIT, as the exact fecal Hb concentration could be of great clinical use. There is evidence that fecal Hb concentration is related to the severity of advanced neoplasia [39]. By adding fecal Hb concentration in predictive models, individuals with the highest risk of advanced neoplasia can be identified [40]. This may also allow for gender-specific approaches. Combining individual fecal Hb concentrations with other risk factors for CRC to base colonoscopy indication on, and not solely a qualitative test result, could possibly improve FIT-screening efficiency. Also, FIT could be of clinical importance after the initial positive test result, because the fecal Hb concentration is associated with the risk of a second colonoscopy within 1 year after screening colonoscopy [41]. Future research in FIT screening should therefore explore the possibilities of incorporating individual fecal Hb levels in CRC screening programs.

## DNA- and RNA-based biomarker tests

DNA- and RNA-based stool tests aim to detect markers of aberrant DNA or RNA from neoplastic cells. They are based on the principle that colorectal neoplasms shed surface cells in stool. DNA or RNA from these cells can be isolated and tested for the presence of mutations and epigenetic changes acquired during carcinogenesis. DNA- and RNA-based testing is relatively new compared to FOBTs. DNA analysis techniques are developing rapidly and are very sensitive [42].

### DNA markers

A recent study combined FIT with several DNA markers, consisting of molecular assays for aberrantly methylated BMP3 and NDRG4 promoter regions, mutant KRAS, and β-actin (a reference gene for human DNA quantity) [43••]. This multi-target stool DNA plus FIT test had a significantly higher sensitivity for advanced adenomas (42 %) and a somewhat higher sensitivity for CRC (92 %) than FIT alone (sensitivity for advanced adenoma 23 %, for CRC 72 %). However, this increase in detection came against the background of a considerably higher test positivity rate (16 vs. 7 %). As a consequence, the demand for colonoscopy is more than twice as high after DNA-FIT testing than after FIT alone, and the DNA-FIT test had a lower specificity for advanced neoplasia compared to FIT, 87 versus 95 %, respectively. In the light of population-based screening and limited colonoscopy resources, the higher positivity rate and lower specificity are important pullbacks. Since colonoscopy resources are in many regions the limiting factor in population screening, it has been advocated to compare non-invasive tests not at a fixed test cut-off, but over a range of positivity rates, allowing a direct comparison between tests at the same positivity rate [19]. Also, the multi-target DNA test requires a full stool sample to be sent in a container, which comes with additional costs and impracticality to an already expensive testing procedure. Furthermore, the multi-target DNA test is recommended once every 3 years, whereas FIT is offered annually in the USA. Cumulative sensitivity, specificity, and costs after 3 years of annual FIT screening would therefore be the fairest comparison before drawing conclusions on superiority of either of the two tests. Lastly, adherence to the multi-target stool DNA test has not yet been investigated. Since adherence is crucial in screening efficacy, this should be evaluated before proceeding to implementation of the test in a screening program.

The performance of DNA tests may differ per CRC subtype, on the grounds that CRC is a heterogeneous disease that can develop via multiple pathways. To detect all CRC subtypes with a screening test, different tumor markers have to be used [44, 45]. A recent study showed that different subtypes are associated with marked differences in survival. Subjects with tumor markers reflecting a serrated morphology have the highest disease-specific mortality (hazard ratio 2.20) [46]. This was compared to subjects with tumor markers reflecting the traditional adenoma-carcinoma sequence (the most predominant tumor). However, the biologic basis for the observed difference remains an important topic for future research.

### RNA markers

A microRNA (miRNA) is a small non-coding RNA molecule (containing approximately 22 nucleotides), which functions in RNA silencing and post-transcriptional regulation of gene expression. MiRNAs are thought to be cell-type and disease-specific and may be quantified in stool by quantitative real-time polymerase chain reaction (qPCR) [47]. Aberrant expression of a specific miRNA may display the effects of a tumor suppressor or oncogene. A large number of studies of single or panels of miRNAs for the detection of CRC have been published recently [48]. A Japanese group showed that the addition of fecal miRNA-106a to FIT testing improved the sensitivity but decreased the specificity of FIT [49]. Since most published studies were based on selected populations, further research in an asymptomatic population should be conducted.

## Protein markers

Protein-based stool markers focus on either the detection of cancer-specific proteins or detection of proteins released from inflamed and/or bleeding tissue. Fecal tumor M2 pyruvate kinase (M2-PK) has received the most attention as a potential cancer-specific protein marker. The test is based on the detection of proteins in stool derived from neoplastic colonocytes. A recently reported meta-analysis of studies comparing M2-PK with colonoscopy reported a pooled CRC sensitivity and specificity of 79 and 80 %, respectively [50].

A non-cancer-specific protein marker is calprotectin. Calprotectin is a calcium-binding protein in granulocytes, macrophages, and epithelial cells. Elevation of calprotectin occurs during intestinal inflammation, including inflammation caused by inflammatory bowel disease. Elevated fecal calprotectin in CRC is suggested to be due to neutrophil shedding from an ulcerated tumor into the intestinal lumen. A large Norwegian CRC screening trial evaluated calprotectin and reported a lower sensitivity and specificity than FIT [51].

So far, no single protein stool marker has shown to be of adequate accuracy to be considered for population-based CRC screening. A Chinese study investigated the possibility of combining seven biomarkers in a biochip for the detection of colorectal cancer [52]. The most optimal result was the combination of two biomarkers (TPO, FGF-23) leading to a sensitivity of 0.8 with a specificity of 0.7 for detection of cancer. The use of protein makers in CRC screening, or combining protein markers with FIT, requires further research.

Currently, novel molecular tests to analyze stool for a combination of genetic, epigenetic, and protein biomarkers are being developed. The largest is the Molecular Early Detection of Colorectal Cancer (MEDOCC) project. It is a long-term collaborative research between the Netherlands and USA.

## Human fecal microbiome-based biomarkers

Recent studies suggest an important role for the gut microbiome in the development of CRC. Patients with CRC have a different gut microbiome than healthy subjects [53]. One of the first bacteria more commonly found in patients with CRC was *Streptococcus bovis* [54]. At present, other bacteria have been identified that play a role in gastro-intestinal cancers, such as *Helicobacter pylori*, and *Fusobacterium nucleatum* [53]. The latter has been of particular interest in colorectal neoplasia, with several studies indicating that *F. nucleatum* in feces is associated with the occurrence of colorectal adenomas and cancer. However, its precise role in this process is poorly understood [53]. It has been suggested that *F. nucleatum* could be useful in detecting serrated polyps [55]. This is relevant as FOBT does not seem sensitive to serrated lesions [56]. The fecal microbiome in CRC screening has largely been unstudied, but measuring the fecal microbiome to identify those at risk of CRC seems promising as a novel screening method. A major advantage of this method of screening could be that non-bleeding lesions are also detected. One study combined the gut microbiome with other risk factors and found that by adding the microbiome, the pretest to posttest probability increased, resulting in better identification of subjects with advanced neoplasia [57]. The use of the gut microbiome as a CRC screening tool has great potential. Hence, studies identifying specific microbiota that are associated with CRC are much awaited.

## Conclusion

There is a wide range of fecal tests for colorectal cancer screening available. The guaiac FOBT was one of the first fecal tests used in colorectal cancer screening. Large trials have shown a significant reduction in CRC-related mortality after screening with gFOBT. However, its use has several limitations when compared to FIT. These limitations include ease of use, adherence, and sensitivity. Also, FIT has the advantage that fecal Hb concentrations can be measured yielding a quantitative test result. Nonetheless, at present, FIT is mostly analyzed using a pre-determined cut-off. Aside from FOBTs, to date, only DNA-based stool tests have undergone the full spectrum of development and testing for clinical practice. The multi-target stool DNA test was approved by the FDA in the USA for CRC screening in 2014. So far, biomarker tests such as the multi-target stool DNA test are more expensive than the FOBTs, and come with a relatively low specificity. Furthermore, adherence rates have not been evaluated. Therefore, a sensitive single biomarker or panel of biomarker (stool) tests at affordable cost is much awaited. Also, further identification of the gut microbiome could open up new possibilities in CRC screening strategies. Expansion of molecular biomarker screening tests may become imaginable in the future. At the current stage, screening by means of FIT seems the way to go. Requirements in test sensitivity, specificity, and costs in order for new molecular biomarker technologies to be cost-effective compared to the FIT should be investigated. Future focus should also be on using FIT quantitatively and incorporating FIT results in risk prediction models to maximize screening benefit and efficacy.

## Abbreviations

CRC: Colorectal cancer
DNA: Deoxyribonucleic acid
FIT: Fecal immunochemical test for hemoglobin
FOBTs: Fecal occult blood tests
gFOBT: Guaiac fecal occult blood test
Hb: Hemoglobin
miRNA: Micro RNA
RNA: Ribonucleic acid
PCR: Polymerase chain reaction
